# Doing Family in “Times of Migration”: Care Temporalities and Gender Politics in Southeast Asia

**DOI:** 10.1080/24694452.2020.1723397

**Published:** 2020-03-16

**Authors:** Brenda S. A. Yeoh, Bittiandra Chand Somaiah, Theodora Lam, Kristel F. Acedera

**Affiliations:** * Department of Geography and Asia Research Institute, National University of Singapore; † Asia Research Institute, National University of Singapore

**Keywords:** care rhythms, gender roles, left-behind children, return migration, temporalities, transnational family

## Abstract

The prevailing labor migration regime in Asia is underpinned by rotating-door principles of enforced transience, where low-wage migrant labor gains admission into host nation-states based on short-term, time-limited contracts and where family reunification and permanent settlement at destination are explicitly prohibited. In this context, we ask how migrant-sending families in Southeast Asian “source” countries—Indonesia and the Philippines—sustain family life in the long-term absence of one or both parents (often mothers). Through temporal concepts of rhythm, rupture, and reversal, we focus on how temporal modalities of care for left-behind children intersect with gendered power geometries in animating transnational family politics around care. First, by paying heed to the structuring effects of rhythm on social life, we show how routinized care rhythms built around mothers as caregivers have a normalizing and naturalizing effect on the conduct of social life and commonplace understanding of family well-being. Second, we explore the potential rupture to care rhythms triggered by the migration of mothers turned breadwinners and the extent to which gendered care regimes are either conserved, reconstituted, or disrupted in everyday patterns and practices of care. Third, we examine the circumstances under which gender role reversal becomes enduring, gains legitimacy among a range of poly care rhythms, or is quickly undone with the return migration of mothers in homecoming. The analysis is based primarily on research on Indonesian and Filipino rural households conducted in 2017 using paired life story interviews with children and their parental or nonparental adult caregivers.

## Abstract (zh)

亚洲现行的劳工移民制度大多都是“反复快进快出”的强制性短期停留, 即低工资的移民劳工通过有时间限制的短期合同进入东道国, 但严格禁止他们在目的地与家人团聚和永久定居。在这一背景下, 我们走访了东南亚劳工主要来源国家（印度尼西亚和菲律宾）的移民家庭, 了解这些家庭成员如何在父母双方或一方（通常是母亲）长期缺席的情况下维持家庭生活。我们通过节奏、破坏和逆转的时间概念, 关注了在跨国家庭政治中围绕照顾留守儿童的问题, 探索其时间模式如何与性别权力架构产生交叉。首先, 我们关注了节奏对社会生活的结构性影响。由于母亲在日常子女照顾节奏中的核心地位, 已经被视为一种惯例, 作者分析了在社会生活行为方式以及对家庭幸福概念的普遍理解中, 人们如何对这种惯例习以为常并视其为自然现象。随后, 我们探讨了由于母亲为养家糊口而去往国外, 可能会打破这种节奏, 并分析了在日常照顾方法和实践中, 基于性别的照顾模式被保留、重建或破坏的程度。最后, 我们研究了让性别角色逆转持续存在的情况, 或者会通过一系列综合性的保育节奏, 将这种情况被合理化；或者在母亲返乡后又迅速恢复原状。该分析主要根据 2017 年对印度尼西亚和菲律宾农村家庭的一项研究展开, 该研究通过配对访问的方式, 分别展示了儿童及其父母或非父母的其他成年监护人的生活。

## Abstract (es)

El régimen dominante de la migración laboral en Asia está respaldado por los principios de puerta giratoria de forzosa transitoriedad, donde el trabajo migratorio de paga baja consigue admisión en naciones–estado anfitrionas, bajo el estilo de contratos laborales a corto plazo y por tiempo limitado, y en donde la reunificación de la familia y el asentamiento permanente en el lugar de destino de la migración están prohibidos explícitamente. En este contexto, en los países “fuente” del Sudeste Asiático preguntamos sobre el modo como las familias que envían migrantes ––Indonesia y las Filipinas–– sostienen la vida familiar durante la ausencia de largo plazo de uno o ambos padres (a menudo las madres). A través de los conceptos temporales de ritmo, ruptura y la reversa, nos enfocamos en cómo las modalidades temporales del cuidado de los niños que se dejan atrás se cruzan con las geometrías de poder por género para darle ánimo a las políticas transnacionales de familia. Primero, prestando atención a los efectos estructurantes del ritmo sobre la vida social, mostramos cómo los ritmos rutinarios del cuidado construidos en torno a las madres como prestadoras de cuidados tienen un efecto normalizador y naturalizante en la conducta de la vida social y el corriente entendimiento del bienestar de la familia. Segundo, exploramos la ruptura potencial de los ritmos del cuidado disparados por la migración de las madres, convertidas en las que ganan el pan, y la extensión con la cual regímenes de cuidado por género son conservados, reconstituidos o perturbados en los patrones y prácticas cotidianas del cuidado. Tercero, examinamos las circunstancias bajo las cuales la reversa del papel del género se hace duradera, gana legitimidad entre un rango de ritmos de cuidado, o es rápidamente deshecha con la migración de retorno de las madres al regresar al hogar. El análisis se basa primariamente en la investigación de hogares indonesios y filipinos conducida en 2017 usando entrevistas de historia de vida por pares, con sus hijos y sus cuidadores adultos emparentados o no.

In the last three decades, scholars interested in social change in Southeast Asia have noted the potentially catalytic effects of an increase in feminized migrant labor flows in response to the growing demand for domestic and care work in more developed countries. The prevailing labor migration regime in Asia is underpinned by rotating-door principles of enforced transience, where the overwhelming majority of migrants—particularly those seeking low-skilled, low-wage work—are admitted into host nation-states on short-term, time-bound contracts, with little or no possibility of family reunification and permanent settlement at their destination. This regime of temporary migration is in turn predicated on sustaining a transborder household division of labor for family formation and maintenance. In this context, the “times of migration” (Cwerner 2001, 9) are experienced quite differently from what is predicted in traditional migration and settlement scholarship, where “the once-transient migrants increasingly become permanent settlers” through transitional processes of assimilation and acculturation and over generational time. As Halfacree (2012) observed in his study on urban–rural circulatory movements, “the idea of ‘permanent’ migration increasingly seems a product of an implicit assumption of normative sedentarist settlement” (213). Focusing on Southeast Asia—the fount of feminized contract-based transnational migration—provides us an opportunity not only to resist such an assumption but to investigate its gendered contours. In this light, we train our analytical lenses on one of the most basic social units of sedentism, the family, as its gender-normative inner workings become subject to “transnational stretch” in response to women’s breadwinning migration.

Split across national borders for prolonged, often unpredictable lengths of time, low-wage migrants and their left-behind family members sustain the “transnational family” through continuous circuits of money, material goods, care, and affection between home and host countries (Yeoh 2015). For many migrant-sending families in Southeast Asian source countries such as Indonesia and the Philippines, an important component of “doing family” across borders relates to negotiations around temporal modalities of care for the well-being of children in the absence of one or both parents. In this context, this article examines *care temporalities*, or how care relations are molded, negotiated, or transformed across modalities of temporal experience linked to the vicissitudes of temporary migration. We make three interrelated contributions to the burgeoning literature on the geographies of migration and the links between social and spatial change. First, whereas Sassen’s (2000) much-cited work has focused on the contradictions between coexisting temporal logics of economic globalization and the nation-state in the crucible of migrant-receiving global cities, we turn attention to the much more neglected context of sending communities in the developing world. Second, we foreground the temporal dimension (alongside the social–spatial) in bringing together the liminal times of migration and the intimate times of the family to gain better insight into the gender politics of care among left-behind families. Third, we attend to multiple temporal modalities that scaffold families in migration as they attempt to reconcile gendered caregiving with increased geographical mobility.

We first build on the growing but still limited scholarship on the temporal dimensions of migration to provide a conceptual framing featuring three temporal concepts: rhythm, rupture, and reversal. This is followed by a discussion of the migrant-sending context in Indonesia and the Philippines as well as the methodological underpinnings of the research. Drawing primarily on life story interviews, the rest of the article shows how the temporal concepts identified can be productively engaged in furthering our understanding of the gender politics at work in caregiving among transnationally stretched families. In the conclusion, we attend to how temporal modalities work to contextualize gender politics around the care of left-behind children and shape the potential for social change.

## The Temporalities of Migration and Family: Rhythms, Ruptures, and Reversals

Mazzucato and Schans (2011) were among the first scholars to argue that “methodologically and theoretically, families are still predominantly conceived of as nuclear, living together, and bounded by the nation state. … Transnational families … have been treated as a temporary phenomenon, with family reunification in the host society the preferred outcome for all family members” (704; see also Mazzucato and Dito 2018). With the increasing ubiquity of the “permanently temporary” migrant in regimes of unflexible noncitizenship in Asia, attention to family morphologies that bear the marks of the seemingly transgressive forces of increased migrations and mobilities has become more urgent. In response, scholars have focused primarily on how transnational families are lived in terms of spatial (dis)connectivities, as witnessed in the burgeoning literature on the complex role of information and communication technologies in negotiating intimacies and other kinds of relationships across transnational space (Peng and Wong 2013). To take one insightful example, Parreñas (2014, 426) differentiated between what she called “intimacy across distance [that] is constructed primarily via routine” (face-to-face communication) and “intimacy in proximity [that] is mainly premised on instantaneity” (information and communication technology–mediated communication). The former is based on careful time management, whereas the latter depends on the privilege of immediate access. Arguing that instantaneity, or “absent presence” (Pertierra 2006), as a feature of intimacy is a gendered expectation tied more to transnational mothering than transnational fathering, Parreñas (2014) urged us to valorize different temporal modalities in performing intimate labor in maintaining “*routine rhythms* of family life” (429). This helps us avoid diminishing, or even demonizing, the intimate work that migrant women do across distance.

Although there have been recent calls to pay greater heed to time and temporal dimensions (Robertson and Ho 2016; Baas and Yeoh 2019), substantive work focusing on critical temporalities in transnational migration research is still limited. Some two decades ago, Cwerner’s (2001) seminal article “The Times of Migration” drew attention to the fact that the potentially disruptive aspects of migration in uprooting and regrounding migrant life are not merely spatial but also temporal. As Zhou (2015) observed, however, transnationalism is largely theorized as “spatiality-based … [foregrounding] the networks and relationships that cross state borders” (163); hence, it is unsurprising that much of the work on the transnational lives of migrants and their families has focused on the styles and strategies of transcending space and negotiating its bordering effects. Geographers, in particular, have noted that in studies of transnationalism, the significance of different modes of “temporality” to transnational processes tends to be overshadowed by a preoccupation with “simultaneity,” where the primary interest is in occurrences at the same time that connect across space (see Huang, Yeoh, and Lam 2008; Waters 2011). The ongoing challenge is to go beyond simultaneity and to theorize “spatiality and temporality in ways sensitive to their mutually constitutive relationship … [in order to] avoid new fixity and hierarchy, and … resonate with a fluidity and fleetfootedness” (Bailey 2009, 413) that condition transnationally linked lives.

Critical interest in the temporal dimensions of transnational migration has resurfaced in recent times, but the bulk of the scholarship has focused largely on host societies as the context for studying the effects of temporal processes on migrants. As forms of trajectory, means of regulation, or signs of disjuncture, temporalities matter in shaping migrant experiences of border crossing, legal processes, work discipline, social networks, care relationships, and spatial mobility, integration, and belonging (Waters 2011; De Genova 2013; Robertson 2015; Zhou 2015; Axelsson, Malmberg, and Zhang 2017). For example, permanent residency permits and dual citizenship statuses accessible to highly skilled migrants facilitate the growth of transnational lifestyles, whereas migrants in the low-skilled and low-paid sectors have no access to (eventual) “permanency” and experience a different kind of transnationality associated with marginality, inequality, and exploitation (Collins 2012). The inherent inequalities of temporary migration programs result in disadvantaged migrants experiencing “various and dynamic subjective senses of precarity, rootedness, pendularity, suspension and nomadism” (Robertson 2015, 51). Temporal periods of immobility characterized by notions of stillness, waiting, and being stuck (Cresswell 2012) become as significant to study as the frequency and fluency of mobility associated with transnational lifestyles.

We thus argue that highlighting time and temporality in transnational space encourages us to understand “migration and its antonym (non-migration) not as contradistinctive phenomena but umbilically conjoined” (Baas and Yeoh 2019, 162). We build on the emerging literature on transnational migration, family life, and critical temporalities to understand the changing temporal modalities of care for left-behind children growing up in the increasingly normalized context of mother migration. In affirming the view that transnational family dynamics are as much concerned with coordinating time as with overcoming space, we depart from the current locus of concern situated in host societies and turn instead to focus on how transnational temporalities work in shaping family life in source communities. To advance our work, we draw on three temporal concepts: rhythms, ruptures, and reversals.

Composed of “repeated moments of movement and rest” (Cresswell 2010, 23), *rhythm* is a social category of time that predominantly foregrounds regularity and order. The rhythmic orderings of everyday life “contribute to how people experience daily life and how they value their own rhythms in relation to those of others” (Lager, van Hoven, and Huigen 2016, 1569). They often confer a sense of order, routine, and recurrence; synchronize collective lives; and provide a basis for anticipating the future as more of the same. By paying heed to the observation that “rhythms are necessarily implicated in the structure and restructuring of social worlds” (Reid-Musson 2018, 883), we focus on how routinized care rhythms built around mothers as caregivers have a normalizing and naturalizing effect on the conduct of social life and widely accepted understandings of family well-being.

As a protracted act of spatiotemporal uprooting and regrounding, migration is a process “inherently characterized by *rupture*—a break, change, distance, division” in everyday life (Boehm et al. 2011, 1). Not only do migrants confront discordances that stem from moving to unfamiliar places and engaging with different regimes of value, but “much of social life is potentially disrupted” (Cwerner 2001, 15) even for left-behind family members. Temporal ruptures connote “time that is punctuated rather than enduring: of fateful moments and turning points … dates as qualitatively different rather than quantitatively cumulative” (Guyer 2007, 416). The superimposition of different temporal grids on the life worlds of migrants and their families often leads to the disruption and fragmentation in the way the past, present, and future are experienced (Acedera and Yeoh 2019). These ruptures, or “rifts between different temporalities,” could “produce ‘perturbing’ effects over the lives of individuals,” who may also experience “difficulties to ‘pass’ from one time to another” (Cwerner 2001, 18). To sustain the family, members have to adjust to interrupted rhythms and synchronize the discrepant temporal orderings of both home and host societies. In Gasparini’s (2004) words, these families attempt to create a “coexistence of different temporal orientations and actions that live together at the heart of a complex temporal architecture” (420). To understand the limits to restitching family in times of migration, we explore the potential rupture to care rhythms triggered by the migration of mothers turned breadwinners and the extent to which gendered care regimes can be conserved, reconstituted, or disrupted in everyday patterns and practices of care.

In migration studies, *reversal* as a social category of time is often associated with the spatial notion of return. Xiang (2013) suggested that “the word *return* establishes the directionality of mobility—directionality in ethical terms instead of only in the physical sense” (16). As return migration—that is, a spatial move back to “origin”—is often perceived as the virtuous completion of a temporary sojourn away from “home,” social relations that constitute the family are also expected to revert to what is thought to be the original (and therefore desired and destined) state. Reversal challenges the view of the migration trajectory as “a linear process of self-development, adaptation and participation” (Cwerner 2001, 18) in the host society. As a temporal experience in the context of return, reversal is often perceived to be restorative, akin to a process that reinstates the “natural” order of things. At the same time, though national frameworks that govern transnational mobility tend to position return migration as having “naturalizing and normalizing effects” (Xiang 2013, 16), the relationship between return and reversal is not causally determined but contingent. Instead, return might also open up possibilities for the rupturing or reembedding of care rhythms, thus producing a concurrency of diverse care temporalities. In this light, we draw on Lefebvre’s (2004) notion of *polyrhythmia* or diverse, coexisting rhythms, to examine the circumstances under which gender role reversal in caring for children gains legitimacy and becomes enduring or is quickly undone with the return migration of mothers in homecoming. We thus follow Cwerner (2001) in claiming that “by revealing the interplay between ruptures and continuities, old rhythms and new routines, a focus on the temporal dimensions of [care] experiences can provide a critical anchor for understanding the process, dynamics and possibilities of … migration” (15) and its mutually constitutive effects on the family.

## Research Context and Methods

In response to the global—if gender-segmented—demand for waged domestic and care labor, developing countries in Southeast Asia have increasingly promoted overseas labor migration as a development strategy to address issues of poverty, domestic unemployment, and underemployment and to grow foreign exchange income through remittances (Asian Development Bank 2012). Women in the region move across transnational space—often as cheap and flexible labor with severely diminished rights—to plug growing care deficits (in terms of child care, elder care, and care for the sick) in globalizing cities and more developed economies.

As Southeast Asia’s pioneer in producing global workers, the Philippines supplies “workers of varying skill levels to over 100 countries” (Asis, Scalabrini Migration Center, Philippine Migrants Rights Watch, and Friedrich-Ebert-Stiftung 2005, 27). Making up 8 percent of the country’s 100.98-million-strong population, the 7,979,716 Filipinos abroad in 2015 is a significant and reliable source of revenue (Department of Foreign Affairs 2015). Personal remittances accounted for 10.2 percent of the country’s gross domestic product in 2018 (de Vera 2017). Among overseas Filipino workers, women migrants make up 55.8 percent of the total deployment (Philippine Statistics Authority 2017). State promotion of the deployment of Filipinos as overseas contract workers is wedded to a discourse of the overseas contract worker as a national hero, an appellation that arises “from a specifically economic calculus and an unquestioned belief in the national developmental potential of [remittances as an] income stream” (Gibson, Law, and McKay 2001, 369). This is accompanied by the development and elaboration of an institutional and legal framework governing all phases of migration, from predeployment to on-site services, to the return and reintegration of migrant workers.

Indonesia has also followed suit in “embrac[ing] the logic of remittance income as a reliable source of external finance” (Rupert and Solomon 2006, 90). Initially, the Indonesian state employed a specific strategy of providing unskilled workers at relatively low cost to give its workers a comparative advantage over the Philippines. The undertaking has been aided by the development of a migration industry that “worked assiduously to increase both the supply of women in Indonesia and in expanding the market of their labor overseas” (Hugo 2005, 70). Today, overseas labor migration has become an important development strand of Indonesia’s economy, where remittances from its 9-million-strong overseas workforce account for approximately 1 percent of Indonesia’s overall gross domestic product, or US$8.9 billion in 2016 (World Bank 2017). Female migrants accounted for 62 percent of the total deployment in 2016, and domestic workers contributed about 51 percent of total remittances (World Bank 2017). Remittances have surpassed official development aid since 2005 (Elias 2013), highlighting the significance of remittances for household poverty alleviation.

In sum, rural households in the Philippines and Indonesia have turned to the logic of remittance income as a means of migrating out of poverty and aspiring to a better life. Families in developing Southeast Asia absorb, process, and act on opportunities or threats posed by major structural change, thus acting as the all-important link between macro and micro factors of socioeconomic change accompanying migration. Noting that migration and family have mutually constitutive effects, we build on the emerging literature on transnational migration, familial care work, and contingent temporalities to understand the changing modalities of care for left-behind children growing up in the wake of parental migration.

The study is based primarily on qualitative research conducted in 2017 (with supplementation from earlier interviews conducted between 2009 and 2012) with Indonesian and Filipino rural households in provinces with high levels of out-migration outside the main metropolitan areas, namely communities in East Java, Indonesia, and Tagalog-speaking communities in the province of Laguna, Philippines. Despite differences in cultural and religious heritage between Indonesia and the Philippines, the study sites share the common characteristic of being located in areas of high out-migration and areas where the evolving material landscapes testify to flows of remittance monies and investments in house-building projects. For this analysis, we draw primarily on 107 paired life story interviews (from the same household^1^) with parental or nonparental adult caregivers and their children in two age groups—about half in middle childhood aged eleven to fourteen and the other half young adults aged seventeen to twenty. At the time of the interviews in 2017, fifteen of those interviewed had fathers who were current migrants, twenty-six had migrant mothers, and fourteen had both parents as migrants. The remaining were a mix of nonmigrant and returned migrant households. We adopt the view that “(m)ethodologies incorporating more than one individual in a household can shed light on the multiple subjectivities within migrant households and foreground the perspectives of young people by treating them as equals” (Dobson 2009, 358). Interviews were conducted in participants’ own homes and in the local language with which they were most comfortable. Children were interviewed within sight of an adult in their household but, where possible, out of earshot to ensure their comfort while eliciting less restrained responses. By journeying “back and forth between young people’s own perspectives and that of their caregivers and/or left-behind or returned migrant parents” (Huijsmans 2014, 295), we give weight to different voices (anonymized by pseudonyms) within the same family to explore discrepancies and alignments that speak to the gender politics in the social provisioning of everyday and generational care sustaining transnationally stretched families.

## Routinized Care Rhythms: Mothers as Caregivers

Prevailing cultural and social norms in Southeast Asia undergirding the household division of labor cast men as breadwinners and women as caregivers. It hence came as no surprise that in the household survey, mothers as caregivers emerged as the normalized pattern of care in nonmigrant households. For the majority (85.5 percent) of nonmigrant Indonesian and Filipino households (*n* = 573) with children in their early teens, mothers are the primary caregivers of their children (compared to 7.7 percent with father caregivers and 6.1 percent with caregivers who are close female relatives). The gender household division of care labor continues to prevail in father migrant households (*n* = 140): Everyday care arrangements for children are anchored by mothers as principal caregivers in 87.1 percent of the households where fathers have migrated.

Despite the growing visibility of a culture of migration featuring women as active participants in our study sites, the rhythms of caregiving and care receiving that structure and reproduce everyday life continue to be predicated on essentialized notions that care work constitutes a mother’s fundamental identity. In the case of Carol (Filipino, age forty-six), despite successfully managing multiple “side” businesses, she still considers herself a “stay-at-home mum” who runs the whole household while her husband works. Espousing the view that it is a mother’s responsibility to do her utmost to ensure the well-being of the household over its life cycle, Carol has been caring for her children and grandchildren, while “assisting” her husband with managing household finances to stretch every peso. She runs her “side” entrepreneurial endeavors from their home, allowing her flexible time in structuring her schedule to fulfill her caregiving roles. Carol’s care rhythms have been so effective that they outlasted the care needs of the nuclear family and unfolded seamlessly into the next generation. The pivotal nature of her routinized care rhythms in sustaining family well-being across generations enables her to effectively threaten when angered, “I will leave all of you! Your children won’t grow healthy and fat!”

Even in cases where women have formal jobs outside the home, gendered care rhythms continue to prevail in many cases. Felicia (Filipino, age forty-three), a teacher, has taken care of “everything”—from cooking, to cleaning, to caring for the children—while her electrician husband worked abroad. She also manages the “cashwork” (i.e., allocating and making do with remittances) as part of her care work, as do many other mothers interviewed in Indonesia who rhetorically “rule the roost” (Brenner 1995) while shouldering the bulk of the responsibility for the family. Felicia’s rhythmic, hands-on, constant care in the household was essential in minimizing the potentially disruptive effects of her husband’s migration on her children. Part of the care routine involves daily orchestration of the transnational family’s long-distance communication rhythms. Felicia designed a schedule allowing her thirteen-year-old son an hour-long nightly video chat with his father in a bid to foster closer bonds between the absent father and son. She demonstrates that the mother’s unremitting rhythmic care work not only maintains household harmony but also indemnifies paternal migration.

Among the majority of left-behind mother caregivers, the gender fixity that underpins care rhythms is linked to the common refrain that men should migrate and women should stay behind. Felicia explained why:

It’s really the men [who should go abroad]. The women are the most powerful in the family so it’s necessary for them to be the person to guide their children [here]. … Women can do multitasking. She can work, she can take care of the family … she can guide the family; unlike with men, they can only work … that’s what I experienced with my husband. It is [impossible] to try switching places. In the first place, men don’t know how to budget, where to take their money! Not like us.

The deeply rooted view that mothers are irreplaceable caregivers and fathers are deficit caregivers was echoed by other participants, including Yanto (Indonesian, age thirty-five), who had assumed care for his younger siblings when both of their parents migrated to work overseas. Despite having successfully provided care for fourteen-year-old Diana (including meticulously supervising her schoolwork), Yanto was of the view that, particularly when children are of schooling age, the mother’s role “cannot be replaced” as “she must be 100 percent guiding her children since the very beginning.” He elaborated:

Most of the women here go to Hong Kong and Taiwan. It’s very bad indeed. Sometimes they are taken care of by their fathers, aunts, or grandparents. But the love can’t be the same as a mother’s love. It’s very bad. Sometimes if the father is the one who goes abroad, and the mother stays at home, [their children] can do well in school. But if the mother is not around, their father would have a tough time. … It’s like when a mother brings her child to school, she will ask the school about her child’s development, and after that she will send the child to tuition … the child will study with the mother. The mother will teach the child. But if it’s a father, then there’s a huge difference. It’s an exception to find a father who really understands about education. Maybe there’s only one in this village. One in one hundred maybe. But he still can’t be like a mother!

Indeed, the impossibility of substituting for a mother’s temporally sensitive and embodied role is rooted in the belief that only mothers would invest in a care routine that would ensure that the child achieves the best possible outcome in school. This logic is also discernible in Supartinah’s (Indonesian, age thirty-five) case, when her husband refused to allow her to migrate for work on the grounds that mothers need to remain to care for the children’s educational needs:

He prohibited me [to migrate] because who’d be the carer of our children [if I left]? He didn’t allow it [children to be cared for by grandmother]. He thought that the children should be taken care of by their parent [read: mother], like, what about their education? If they’re under the responsibility of their grandmother, perhaps she won’t give attention to their education.

Careful temporal surveillance of children’s studies was considered crucial and the domain of more “up-to-speed” mothers rather than grandmothers, who are thought to be too steeped in past tradition. In Titin’s (Indonesian, age thirty-seven) case, she returned from working overseas the moment her son enrolled in primary school:

It’s time to teach the children. I’m afraid that they will be affected by the negative impact if I [am away]. … I’m afraid of that, especially for [my] son, … of course I want, I want to go again [to earn more money]. But to think that the children will grow up … [and] there’s nobody to watch over them, I’m scared that their mental [state] will be affected.

Care for children is understood as time dependent, with specific windows, because specialized care cannot be delayed, rescheduled, or substituted by remittances. Watching and looking after children requires an embodied, emplaced mother’s constant and keen eye. Although maternal care rhythms can be temporarily suspended when the children are preschool age (and thought to be too young to learn), mothers’ absence is no longer excusable when children reach the school-going, learning phase. Discordant rhythms (or what Lefebvre [2004] called *arrhythmia*) that disrupt normalized routines are thus considered acceptable only when temporary.

Routinized care rhythms built around mothers as caregivers thus have a normalizing and naturalizing effect on the conduct of social life and widely accepted understandings of family well-being. These daily rhythms are based on, and in turn reproduce, essentialized notions of the superiority—and hence indispensability—of mothers’ care for their own children. Attentiveness toward children’s multifaceted care needs as they enfold over the life course—and in particular guiding children through the academic hurdles and moral hazards of school life—requires a consistency and constancy in the temporal ordering of care provisioning that is the assumed strength of mothers, and only mothers.

## Ruptured Care Rhythms? Mothers Turned Migrant Breadwinners

Although the gender-normative mother caregiver model has considerable discursive durability at our study sites, women are also increasingly exposed to the countervailing pull of migration, buttressed by the promise of improving the socioeconomic status of their families and, particularly for mothers, the desire to build up resources to provide their children with quality education. Despite the precarity of overseas contract work, women’s labor migration is often seen to be necessary and inevitable to sustain visions of the newly emerging bourgeois consumerist family (Silvey 2006). When mothers migrate abroad to become primary breadwinners, routinized care rhythms predicated on mothers as caregivers are potentially—but not necessarily—ruptured. The survey shows that in households where mothers migrate to become overseas workers (*n* = 94), primary care for the children is partially redistributed across the gender divide to fathers (57.5 percent), with a third (34.0 percent) passing on the care work to close female relatives such as grandmothers and aunts.^2^ In this context, we discuss three possible care trajectories associated with the temporal rupture of mother caregiver routines as a result of maternal migration: conservation, reconstitution, and irreversible rupture.

### Gender Conservation of Care Rhythms in the Face of Rupture

We begin with a case that illustrates the conservation power of long-distance mothering, where despite prolonged maternal absence, the care relationship continues to strengthen over the child’s life course. Here, we highlight how the temporal aspects of care—as caring needs shift and transform during children’s transition to young adulthood—are closely entwined with generational and gendered politics of who should provide proximate care in the absence of the mother. Kathy’s (Filipino, age eighteen) mother migrated to work abroad when Kathy was thirteen. Her parents had separated a few years before her mother’s migration, and Kathy went to live with her maternal grandparents. Her seventy-two-year-old grandmother, Alicia, who had provided supplementary care for Kathy in her formative years, became her primary caregiver in her mother’s absence.

For a stint, Kathy moved to stay with her father, but this did not last long, and she moved back to her maternal grandmother’s place. As Alicia explained:

[Kathy’s mum] asked me that I should not let Kathy go to her father anymore. … She’s already too grown up to be sleeping there with her father. … There are so many cases of incest now. I was just trying to protect her from what might happen. You cannot tell, sometimes if the father is too drunk, he might lose his right frame of mind.

The transition from childhood to young adulthood entailed changes in care needs, particularly the shift from physical care to more emotionally inflected care relationships. When Kathy was younger, she had a close relationship with her grandmother. At the same time, Alicia was careful to highlight that her own position in Kathy’s life was only as a support to the migrant mother and not a substitution. This was also borne out in our interview with Kathy, who said that although she feared losing her mother when she left, she had over the last few years cultivated a “super close” relationship with her mother. In her words, “Even though Mummy is so far away, still we are super close. We are open with each other [and] always tell each other about the problems that we go through.” She was also appreciative of the fact that her mother’s remittances were instrumental to smoothening her educational pathway to attaining a computing diploma.

Although her mother’s migration was a significant turning point that instilled fear and loss at that point in Kathy’s childhood, the care rhythms that bolstered her well-being were sufficiently conserved so as to modulate any sense of permanent rupture. Despite the separation of time and space, Kathy’s care relationship with her migrant mother strengthened over the years as mother and daughter learned to share intimacies, as well as the hope of eventual reunion. Paradoxically, as she sought more comfort and support from her migrant mother, her relationship with her grandmother had attenuated as she reached young adulthood, primarily because of conflicts over some of her choices (Alicia did not approve of her boyfriend, decision to change religion, and coming home late). Kathy’s strongest desire was to migrate and reunite with “her family” (referring to her mother and her mother’s boyfriend).

Although Kathy had grown to depend less and less on her grandmother for emotional support as she became older, her grandmother provided a constant and reliable pattern of care that had a leveling effect over the turbulence of life course conjunctures such as her parents’ separation and her mother’s migration. It was also her grandmother’s gendered care that served to protect her from possible sexual danger posed by predatory men (including possible harm from a drunken father), although this form of watchful care became less appreciated as Kathy attained young adulthood.

In sum, despite the potential rupture stemming from migration, the gendered care regime—based on a synchronized combination of grandmaternal and maternal care in this case—was largely conserved even as care rhythms evolved over Kathy’s life course. Both the primary care relationships, as well as the normative gender order, were conserved through a feminized restitching of in situ and long-distance care, smoothing over temporal rifts in her adolescent life course.

It should be remembered, however, that redistributing care work to grandmothers often entails “multiple exploitations, including self-exploitation, of women as unpaid caregivers, and further gender inequalities embedded in their temporal ‘flexibility’” (Zhou 2015, 168). Neither is it the case in every circumstance that grandmaternal care becomes the unassailable solution to effect seamless care transitions when mothers migrate. Kusuma (Indonesian, age sixty-five), a widowed grandmother and caregiver of two teenage grandsons left with her from a young age by their serial migrant mother (their father lives in the same household but contributes little to care work), revealed that she struggled constantly with housework (“No one else will clean the house other than me … that is why the house is dirty like this. The boys don’t help clean the house. They don’t want to wash their own clothes”). Kusuma was time-pressed and time-stressed to shoulder the burden of household chores in her older years. She worried unceasingly about the poor school performance of her grandsons, particularly the errant behavior of the older grandson, who blatantly asked her for money to buy cigarettes. Our interview with the younger grandson, Rusli (age fourteen), also revealed growing fissures in the mother–son relationship: Rusli did not find it necessary to save his mother’s number in the mobile phone she had gifted to him by his mother and instead actively distanced himself from her, such as by refusing to sleep in the same bedroom during her home visits. Although the conventional gender architecture of care was conserved by replacing maternal with grandmaternal care, care rhythms appeared to have been compromised, leaving an anxiety-ridden care vacuum around both mother–son and grandmother–grandson relationships.

### Gender Reconstitution of Care Rhythms

As survey findings indicate, when mothers migrate, principal care for left-behind children passes mainly into the hands of fathers (57.4 percent of the cases). Against the commonly held perception of delinquent left-behind fathers who are resistant to stepping up to care duties, recent studies show that they often struggle to create alternative versions of good fatherhood by repackaging masculine identities based on performing care work while retaining a semblance of economic autonomy (Hoang and Yeoh 2011; McKay and Lucero-Prisno 2012; Lam and Yeoh 2018). Although there are men in the study who resist care work in the absence of their breadwinning wives (e.g., Rusli’s father), an increasingly common—and often heroic—refrain that we encountered focused on the way left-behind men rose to the occasion. Fathers as caregivers are still novel enough to attract interest and comment.

Harold (Filipino, age thirty-two) returned from Taiwan to look after his two children when his wife decided to migrate to work in Italy. Although the children’s maternal grandmother, Thelma, was available to lend a hand, he did not want to leave the caregiving solely to her. Thelma had high praise for Harold, calling him “the best father carer” and grading him “100 percent as a father and husband.” She took pains to describe the meticulous nature of his care routine:

After he arrives home from work, he takes care of the children. After washing the clothes, he will attend to the children … look into their needs. He is fond of checking the children’s school materials from books to notebooks. Everything must be in order. He even checks candy wrappers inside the bags. He makes sure that the bag is clean and tidy and that the ballpoint pens are working every day. Even if he is working, he does not neglect his children.

Harold was well aware of the challenges in going against the grain of the prevailing gender order and tried to shrug off the disdain he encountered:

For the children’s clothes worn on special occasions, I wash them myself. [When neighbors laugh at me when they walk past] I [splash water on] them. Besides, people outside are now accustomed to seeing me do that. If they see me washing the clothes, they will be asking me, “Don’t you have work today?” [They think a father should only] work and erm … discipline kids. [But it’s okay to do the housework]. [I prepare breakfast], everything, even underwear [for the children].

He also had to cope when his daughter started menstruating:

Yes, I buy napkins [for her]. I just buy sanitary pads at the store nearby. I feel embarrassed when I buy that downtown, but at the neighborhood store I say, “One packet,” they know what I mean. But I didn’t panic [when Hazel told me about her menstruation], it is normal for a girl.

When fathers assume primary caregiving roles for their children in the absence of breadwinning mothers, a significant reconstitution of gendered care rhythms comes into effect. Unlike mother caregivers, who are full-time housewives, the rhythms of care work for left-behind fathers are often intertwined with some form of income generation, even if this is nominal as compared to the remittances sent home by breadwinning mothers. Jaime (Filipino, age fifty), a tricycle driver and father of three children, took on domestic and caregiving duties when his wife migrated to work abroad ten years ago. He described the interpellation of care work and income-generating work in the repetitive shaping of everyday rhythms:

I wake up at six and leave them their breakfast [in the morning]. I drive my tricycle [for a few hours to earn some income]. At noon, I return home, prepare them lunch, then leave again [to drive tricycle]. In the afternoon, I prepare dinner. … Washing. Cooking. Cleaning the house. Care for the children.

The flexibility of his work schedule meant that Jaime was able to orientate his daily routines toward the care of his children. When his son Jeremy entered high school and became rebellious and joined schoolmates in playing truant, Jaime took action and transferred Jeremy to a nearby school to keep a close watch over him (“Sometimes, I pass by his school [on my tricycle]. I make sure he attends class”). He also policed (again on his tricycle) traditional masculine sites of “vice” such as potential smoking and drinking corners. His attentive modulation of care rhythms earned him his wife’s trust, which he reciprocated by assuming full responsibility for his children, saying, “If something ever happens, I should be responsible, not others … because it’s difficult if you blame others, and your wife will blame you and say, why did you leave your children?”

In college, Jeremy (age nineteen) held progressive views about his parents’ reconstituted gender roles (homemaker father, breadwinner mother). Against the grain of popular opinion, he felt that his mother was the parent who was better at undertaking migration because she was “more capable at being successful abroad.” Conversely, he considered “[his] dad as more caring” in tending to him when he was sick: “Like, he won’t sleep until I have fallen asleep. He will wait until I’ve fallen asleep. With my mum, after she has given me my medicine, that’s it.” For Jeremy, his father’s care work was central to family well-being (“At home, our dad supports us all the time. Sometimes when my dad isn’t at home, the house would be chaotic”).

To summarize, in the face of the potential rupture when mothers turn breadwinners for the sake of the family, gender roles undergirding the normative care regime could be reconstituted in such a way as to allow fathers to repackage good fatherhood to redeem their masculine selves. This tends to be the case when the importance of doing family trumps doing gender for both the migrating mother and the left-behind father. In other words, both parents, with the support of other extended family members, are willing to collaborate in upholding the reversal of roles—at least temporarily—to further the family’s socioeconomic mobility project. In their reconstituted roles as caregivers, fathers like Harold and Jaime express a sense of pride when they can claim that their children are doing well under their charge. These fathers as caregivers construct masculine selves that are anchored on their ability to surmount numerous obstacles to ensure that they are both “successful providers of material welfare” and effective caregivers of their children (Lam and Yeoh 2018, 113).

### Irreversible Rupture

In a minority of cases among our interviews, the gender reconstitution of care for left-behind children spiraled into a breakdown of family relationships and led to more permanent forms of rupture. Winnie (Filipino, age eighteen) was left behind when she was three months old, when her mother migrated as part of a family project to generate remittances for a better life, including saving up for Winnie’s education. Her father, Felix (age forty-three), assumed “hands-on” responsibility of caring for her with the help of her paternal grandmother while undertaking multiple part-time jobs. In his words, “She [Winnie] was given to me and my mother.” According to Felix, when his wife’s family saw that she was remitting funds to him to invest in house-building, they grew envious and started gossiping and spreading false stories to “put him down,” insinuating that he had no right to his breadwinning wife’s remittances. His in-laws accused him of “stealing their daughter” and he became “riled up” when his wife was “brainwashed” into believing “this and that” (i.e., aspersions that he was a failure as a husband who did not contribute financially to the household). This interference with the couple’s marriage and family life led to their separation some seven years earlier. Although he still had thoughts of trying to mend the marriage, he was also pessimistic about the chances of success given the distance between them due to migration. The couple has since ceased all communication.

Winnie, too, was of the view that migration contributed to the dissolution of her parents’ marriage. Echoing her father’s views, she pointed to how gossip, rumor-mongering, and other people’s interference created a wedge in the marriage that grew insurmountable, especially because her parents were physically apart. She was clearly closer to her father and appreciated the fact that her father was there for her at every milestone. For example, when she reached menarche, Felix explained to her what her mother used to do; as she approached young adulthood, he was there to help her handle boyfriend issues. She added that her father used to drink heavily, “but not anymore.” In contrast, Winnie’s relationship with her migrant mother appeared to be full of contradictions. She blamed her mother for giving up on the marriage and felt it “humiliating” to have a broken home. Although she depended on her mother to send her money for tuition fees and school expenses each month (her father’s income was only sufficient for household subsistence) and expected material gifts from her mother, she also resented her mother for making her feel that their relationship was always just about the money (“It feels like she’s paying me [to be her daughter]. … It’s like she thinks I just want money”). Her mother tried to call her three times a week, but Winnie did not reciprocate (“When I have nothing else to say, I just ignore her”). Even when her mother was home between work contracts, Winnie refused to stay with her, choosing to remain with her father.

Without a supportive web of care woven by extended family members, left-behind husbands like Felix seem particularly vulnerable to insinuations that they are undeserving beneficiaries of their migrant wives’ earnings. His dedication to the care of Winnie as a father was not sufficient to dispel the suspicion that he was incapable of earning an income and had to live parasitically on his wife while depriving her natal family of her remittances. Maternal migration thus had a rupturing effect in this case, not just on the marriage but on Winnie’s care relationship with her mother. Despite her mother’s long-distance efforts to provide material care and communicate emotional care, Winnie appeared to be curtailing relational ties with her absent mother in favor of a deepening attachment to her father. In this case, maternal migration and the ensuing gender reconstitution of breadwinning and caring roles in the family led to a form of temporal rupture with more permanent effects on care rhythms, demonstrating how members—including left-behind children—might choose to maintain emotional and material attachments of varying degrees of intensity with certain kinspeople while opting out of transnational relationships with others.

## Reversal in Care Rhythms? Mothers in Homecoming

As seen in the last section, migration as a form of temporal rupture breaks with prevailing care rhythms, leading to the opening up of new possibilities in care temporalities for the future. At the same time, rupture does not completely obliterate the continuity of socially reproduced rhythms (Spurk 2004). Whereas some rhythms can be reinstated through a process of reversal on the return of the migrant, there is also space for the emergence of multiple rhythms based on “the realization of a new social situation, first envisaged as a possible future and then achieved through subjects’ actions” (Spurk 2004, 46). From the survey, in Indonesian and Filipino households where migrant mothers have returned (*n* = 115), the work of caring for the children reverted to the homecoming mother in the large majority of households (72.2 percent). This is in contrast to 13.0 percent of such households where fathers remained the main caregivers and 14.8 percent where close female relatives continued as the main caregivers. We explore next both instances of reversal and diversification in care rhythms in the case of homecoming mothers.

### Reversal and the Restoration of the Normative Gender Order

Wanti (Indonesian, age forty-five) had been working abroad in domestic and care work since her children were young. At the time of her first migration, her husband lost his job and took care of the children, but when their oldest daughter turned thirteen, she assumed care for her younger siblings, because her father was by then busy working, making and selling food at the market. On Wanti’s return more than three years ago, she had wanted to find paid work locally to supplement the household income because the family was struggling financially, sometimes unable to afford rice and other necessities. She found it inevitable, however, that homecoming also meant returning to the domestic sphere, because her mobility outside the home was circumscribed by her husband’s work priorities (even though his work did not adequately sustain the family’s needs). She said in resignation,

What I want is, actually, to work in a city, and come back … go off in the morning, come back in the afternoon, as a domestic helper, washing clothes or something. That’s what I want, but my husband said I can’t. Because I can’t ride a motorbike, that’s the first reason. Secondly, my husband is busy. Because he’s busy, if he were to bring me around he’ll be tired. Because he’s working. He doesn’t have the time. … [Besides] the house will be messy. … So we decided [that I] just to stay at home, taking care of the children.

Although Wanti wanted to continue working outside the home and earn an income, she soon discovered that men’s and women’s time is prioritized differently, revealing the gendered power that men have in protecting their own time at the expense of that of their wives in patriarchal societies. Men’s time—particularly work time—is framed as scarce and valuable, whereas women are expected to work around their busyness. In contrast, women are expected to spend their “busy” time at home sustaining care rhythms for fear that the house might become neglected due to the lack of women’s timely attentiveness to care. Perhaps as further justification for her acquiescence, Wanti continued:

For guys, after they come back home from working, they’re tired and they [do not like that] their meals are not taken care of.

Properly timed and carefully prepared meals, symbolic of a mother’s vigilant care, were foregrounded in this household. Wanti’s children were equally adamant—for different reasons—that she stayed home. When she told her oldest daughter, “Just let me work, you just stay with your siblings,” the reply from her daughter was, “No, I’m tired taking care of them at home. You just stay at home. … I’m already an adult, I want to [leave home for a nearby town] and have my own money.” It is clear, then, that youthful labor power takes precedence over the work aspirations of women who are already mothers and therefore should bear the brunt of domestic responsibilities. Wanti’s youngest son also forbade her from engaging in work away from home because he would then not be able to enjoy his mother’s cooking. As Wanti said in frustration tinged with pride, “He said if I’m working and don’t come back home, he will not have a side dish in his meal [laughs].” From an income-generating mother with control over her personal labor time, Wanti reverted to being a mother whose time was subjected to the care demands of her children and spouse.

For mothers like Wanti, despite long episodes of successful breadwinning abroad, homecoming has incontrovertibly imposed mother-centered care rhythms in gender-regressive ways. Family members—husbands, sons, and daughters—conspire to return the care routine to wives-cum-mothers on their homecoming, as if the breadwinning mother is an aberrant figure, one who is only temporarily absent from home. It is as if family disorder and discomfort—a messy house, a meal with no side dishes, tired and busy family members—can be restored to their ideal state only if the returning mother reverts to her “rightful” place as the linchpin essential for the proper functioning of the domestic sphere. Even when children can detect no difference between the care provided by fathers and mothers, children’s joy at having their mothers home as well as mothers’ longing for their children can collude to channel women’s feelings toward restoring the natural order of things, rendering them complicit in fixing gendered care rhythms.

### Reversal with a Difference

As argued, mothers in homecoming have generally found return to connote re-domestication despite having proven themselves as capable and successful breadwinners abroad (albeit in mainly care and domestic work). A reversal in gender roles back to the “original” state appears to be welcomed as a form of relief, if not expected as restoring normalcy, not only among fathers but also children, even when fathers have previously performed caring duties to good effect. Reversal is also often accepted by returning mothers who try to make up for their physical absence from home and long separation from their children by spending quality time with their children and taking over household chores. Despite these pressures to restore mother-centered care rhythms, return opens up the possibility of polyrhythmic care temporalities as many returning migrant women struggle with redomestication and cherish aspirations to be able to engage in paid work again. In this time-space of negotiation, we note two possibilities for diversification beyond the norm.

First, even where the normative gender order in care provision is restored to the household, care webs tend to feature more active participation from other family members on mothers’ return. Although Gracie (Filipino, age forty) assumed primary responsibility for care and household work on her return (“My husband says, [now that you are back], ‘I don’t do the laundry, it’s yours.’”), she also noted that her husband helped her with the cooking and the cleaning, and continued with ferrying their thirteen-year-old daughter to and from school. In Harum’s (Indonesian, age thirty-four) case, both spouses worked out a practical coparenting division of labor after her return. Her thirty-eight-year-old husband, Hanif, confirmed that he played an active if subsidiary role with the housework and preparing the children for school, saying, “Of course [I would still help my wife with housework] because we both are here. … [Before] it was me alone looking after the children, but now it is both of us.” In other words, a hands-on dual investment of care time between parents might feature amidst the emergence of diverse care temporalities.

Second, it is important to note that for some returning mothers who are resuming responsibility for care work, they do so differently. This is particularly the case if the returning migrant has generated sufficient economic wherewithal to invest in a business, house-building, or farmland or if her children have grown up and achieved academic or career success and are able to contribute to the family’s well-being. In other words, the successful accumulation of social and economic capital through migration provides some women with alternative pathways of return that allow them to avoid being completely reabsorbed into the domestic sphere. Juriyah (Indonesian, age fifty), for example, not only took over the running of her household on return from a long sojourn abroad but also used her savings to open a small business. Lani (age nineteen), the youngest of her children, was grateful to her “sacrificial” mother for making it possible for her to enroll in university and was also heartened to note her mother’s transformation in combining self-care and care for her family. She observed that Juriyah gained “modern” cooking skills and ensured that the food she served was nutritious and yet not too expensive. At her family’s request, she was able to prepare Western and Arab dishes as part of her food work at home. Using photographs taken when she was in domestic service for inspiration, Juriyah also expressed creativity by adapting the design of her former employer’s home when upgrading her current home and kitchen to more “modern” standards in keeping with the times. Lani noted that Juriyah fashioned herself as a modern mother—she wore makeup and did not veil her hair (unlike Lani), even when she was in the public gaze fronting her shop. In fact, Lani said that her friends all commented that Juriyah looked younger and more fashionable than Lani herself. Said Lani in jest, “Clearly, I lost. Can you believe when my friend and I hang out here, she was like surprised, ‘Is that your mum? How come she is younger than you?’” Lani also observed that her return migrant mother exuded a sense of financial and emotional security:

Before going abroad last time, she would be [constantly] thinking of money, economy, family. [On her return] her mindset is calmer, maybe because the children have matured. But mum herself has [become more confident], having her own activity like opening the shop.

Time was now on Juriyah’s side in terms of care work as her grown-up children were less demanding of her care. In sum, for some homecoming mothers, redomestication on return does not mean a reversion to their premigration roles. They can at least depend in varying degrees on the care rhythms established in their long absence, even if these rhythms have grown weaker on their return or are in residual form. For some, where migration has conferred economic security and social capital, care temporalities have also diversified to encompass a broader repertoire of skills from modern cooking techniques to running small businesses. In the potential for change within the temporality of return (Bastia 2011; Girma 2017), these women have been able to reconcile past and present trajectories of the self in effecting self-transformation while caring for their families.

## Conclusion

In commenting on “the invisibility of temporality in present transnational studies,” Zhou (2015) observed that “we know little about how … different temporal spheres associated with transnational care intermingle, collide and reshape family decisions and relationships in their everyday lives, and how both time and place/space play roles in (re)producing and combating inequalities in the context of international migration and transnationalism” (169). In responding to this observation, we have adopted a critical temporalities approach based on “the recognition of the multiplicity and unevenness of social time” (Parkins 2004, 367), giving weight to the “temporalities … at stake during symbolic struggles” (Spurk 2004, 42). More specifically, we have focused on the gendered (and, to a lesser extent, generational) power geometries threaded into the transnational family politics of care in exploring the transitions and transformations in terms of the naturalizing effect of rhythm, the potential for change in rupture, and the multidirectionality of reversal. We show that not only do we need to pay heed to multiple temporal modalities underlying the care work that sustains transnational families but each “temporalized space” constitutes “an observable property of timespace and in this respect provides a tool for the observation of the unobservable” (Lefebvre et al., as cited in Mulíček, Osman, and Seidenglanz 2015, 307–8). In other words, care temporalities in their observable form as rhythm, rupture, and reversal allow us to grapple with underlying social change resulting from the impact of migration on the family.

In this article, we have trained our analytical lenses on the left-behind family at the southernmost end of the care chain in bringing to light what is often understudied in the geographies of migration. Our work shows that an understanding of the impact of migration on care rhythms supporting the well-being of left-behind children requires not only an examination of transnational dynamics influencing “who migrates” but also an understanding of the gendered microfamily and community context affecting “who cares.” By intersecting gender-based inequalities around care with geography-based inequalities around work, time-limited transnational labor migration undertaken by Southeast Asian women has served to “further diversify and complicate temporalities embedded in care practices and relationships” (Zhou 2015, 169). Despite the potential inherent in the increased feminization of labor migration in transforming the patriarchal gender order in families, our conclusions show that there are real limits to transformation, rooted not only in the disempowering effects of the institutionalized temporary migration regime on families and women, but also in the embeddedness of gender-normative care temporalities in the family. At the same time, temporary reversal of roles and new care polyrhythmia have also become evident in the structure of social life in Southeast Asia, particularly when socioeconomic mobility projects are prioritized as collaborative family endeavors. Whereas Spurk (2004, 43, 46) observed that “rapid social change of the surface and appearance of society conceals continuity and social reproduction” (with which we agree), we also claim the reverse: that the seeming lack of observable change in the gender order in care temporalities among migrant-sending communities also masks competition from other emergent temporalities. By attending to different forms of temporality that modulate care work—routinized, ruptured, and emergent—in sending communities, we foreground the significance of the temporal as “a tool for the observation of the unobservable.”

## Notes

1 These households were purposively selected from a large-scale survey of about 1,000 households in each of the two study countries conducted in 2016 to take into account a mix of migration categories (i.e., mother migrant, father migrant, both parents migrants, and nonmigrant households), the child’s gender, and parents’ marital status. Surveys covering various aspects of the household including care arrangements, migration and remittance histories, and children’s well-being were administered to two or three members of the same household. We do not dwell on the surveys in this article but, where relevant, we reference the surveys’ broader findings to support our arguments.

2 The small remainder reflects caregivers such as male relatives and paid domestic workers.
